# Psychotic experiences and subjective cognitive complaints among 224 842 people in 48 low- and middle-income countries

**DOI:** 10.1017/S2045796018000744

**Published:** 2018-12-26

**Authors:** A. Koyanagi, B. Stubbs, E. Lara, N. Veronese, D. Vancampfort, L. Smith, J. M. Haro, H. Oh, J. E. DeVylder

**Affiliations:** 1 Research and Development Unit, Parc Sanitari Sant Joan de Déu, Universitat de Barcelona, Fundació Sant Joan de Déu, Barcelona, Spain; 2 Instituto de Salud Carlos III, Centro de Investigación Biomédica en Red de Salud Mental, CIBERSAM, Madrid, Spain; 3 Physiotherapy Department, South London and Maudsley NHS Foundation Trust, Denmark Hill, London, UK; 4 Department of Psychological Medicine, Institute of Psychiatry, Psychology and Neuroscience, King's College London, London, UK; 5 Faculty of Health, Social Care and Education, Anglia Ruskin University, Chelmsford, UK; 6 Department of Psychiatry, Universidad Autónoma de Madrid, Madrid, Spain; 7 National Research Council, Neuroscience Institute, Aging Branch, Padova, Italy; 8 Geriatrics Unit, Department of Geriatric Care, OrthoGeriatrics and Rehabilitation, E.O. Galliera Hospital, National Relevance and High Specialization Hospital, Genova, Italy; 9 Department of Rehabilitation Sciences, KU Leuven, Leuven, Belgium; 10 KU Leuven, University Psychiatric Center KU Leuven, Leuven-Kortenberg, Belgium; 11 The Cambridge Centre for Sport and Exercise Sciences, Department of Life Sciences, Anglia Ruskin University, Cambridge, UK; 12 University of Southern California, Suzanne Dworak-Peck School of Social Work, Los Angeles, CA, USA; 13 Graduate School of Social Service, Fordham University, New York, NY, USA

**Keywords:** Cognition, epidemiology, low- and middle-income countries, psychotic experiences

## Abstract

**Aims:**

Cognitive deficits are an important factor in the pathogenesis of psychosis. Subjective cognitive complaints (SCCs) are often considered to be a precursor of objective cognitive deficits, but there are no studies specifically on SCC and psychotic experiences (PE). Thus, we assessed the association between SCC and PE using data from 48 low- and middle-income countries.

**Methods:**

Community-based cross-sectional data of the World Health Survey were analysed. Two questions on subjective memory and learning complaints in the past 30 days were used to create a SCC scale ranging from 0 to 10 with higher scores representing more severe SCC. The Composite International Diagnostic Interview was used to identify past 12-month PE. Multivariable logistic regression and mediation analyses were performed.

**Results:**

The final sample consisted of 224 842 adults aged ⩾18 years [mean (SD) age 38.3 (16.0) years; 49.3% males]. After adjustment for sociodemographic factors, a one-unit increase in the SCC scale was associated with a 1.17 (95% CI 1.16–1.18) times higher odds for PE in the overall sample, with this association being more pronounced in younger individuals: age 18–44 years OR = 1.19 (95% CI 1.17–1.20); 45–64 years OR = 1.15 (95% CI 1.12–1.17); ⩾65 years OR = 1.14 (95% CI 1.09–1.19). Collectively, other mental health conditions (perceived stress, depression, anxiety, sleep problems) explained 43.4% of this association, and chronic physical conditions partially explained the association but to a lesser extent (11.8%).

**Conclusions:**

SCC were associated with PE. Future longitudinal studies are needed to understand temporal associations and causal inferences, while the utility of SCC as a risk marker for psychosis especially for young adults should be scrutinised.

## Introduction

Psychotic experiences (PE) are attenuated forms of psychotic symptoms (e.g. delusions and hallucinations) that do not reach the clinical threshold for a psychotic disorder diagnosis. PE are known to be highly prevalent in the general population with a systematic review reporting a lifetime prevalence of 7.2% (Linscott and van Os, 2013) – a figure that is approximately double that of broadly defined psychotic disorders (Perälä *et al*., 2007). It has been reported that PE are transitory in about 80% of the cases, while approximately 7% eventually develop a psychotic disorder (van Os and Reininghaus, 2016).

One of the most consistently reported risk factors for schizophrenia is cognitive decrement in youth (Aylward *et al*., 1984), consistent with neurodevelopmental theories of schizophrenia aetiology (Fatemi and Folsom, 2009). Despite the evidence suggesting that objective deficits in cognition (e.g. assessed using standardised neuropsychological testing) are also often evident in the at-risk mental state (Fusar-Poli *et al*., 2012) or PE (Mollon *et al*., 2016), little is known about the important concept of subjective cognitive complaints (SCC) in people with PE. SCC are everyday memory and related cognitive concerns expressed by people who may or may not have deficits on objective testing, and are common in all age groups (Begum *et al*., 2014). SCC are critical in identifying subtle changes in everyday functioning that are often a precursor for more serious cognitive decline and functioning, which may not otherwise be detected (Hohman *et al*., 2011). Studying the association between SCC and PE is important as the subjective nature of SCC makes it a much more likely candidate for rapid clinical assessment of individuals who may be at risk for psychosis, given that SCC can be self-reported and does not require burdensome neuropsychological testing and specially trained staff. Furthermore, the prevalence of objective cognitive deficits usually increases with age and is most common at older ages when psychosis onset is not common. However, in the case of SCC, previous studies have shown that the prevalence of SCC may be quite similar in younger and older people (Begum *et al*., 2014). This may be because cognitive decline can be very concerning to the individual at younger ages, whereas at older ages, this may be considered normal. Thus, it is possible that SCC may be a particularly useful marker for psychosis risk among younger people who are at higher risk for psychosis.

Furthermore, another notable gap in the literature is the scarcity of data on cognition and PE from low- and middle-income countries (LMICs) as previous studies on this topic, which have used objective measures of cognitive function, are all from high-income countries. This is an important omission as the effect of cognition on psychosis is likely influenced by environmental and genetic factors that may differ between countries with different ethnic compositions or levels of socioeconomic development. In addition, cognitive decrements in individuals in LMICs may be more likely to reflect early static injury than trajectories of postnatal development as factors such as foetal hypoxia, maternal infection and obstetric complications (Barnett *et al*., 2012) may be more common in LMICs (James *et al*., 2018).

Given the lack of studies specifically on SCC and PE, and the scarcity of data on PE and cognitive function in adulthood, especially from LMICs, we analysed predominantly nationally representative community-based data on 224 842 individuals aged ⩾18 years from 48 LMICs, who participated in the World Health Survey (WHS) to (a) examine the association between SCC and PE by age groups; and (b) to assess the extent to which various factors explain the SCC–PE association.

## Methods

### The survey

The WHS was a cross-sectional, community-based study undertaken in 2002–2004 in 70 countries worldwide. Details of the survey are provided in the WHO website (http://www.who.int/healthinfo/survey/en/). Briefly, data were collected using stratified multi-stage random cluster sampling. Individuals aged ⩾18 years with a valid home address were eligible to participate. Each member of the household had an equal probability of being selected by utilising Kish tables. A standardised questionnaire, translated accordingly, was used across all countries. The individual response rate across all countries was 98.5% (Nuevo *et al*., 2012). Ethical approval to conduct the study was obtained from the ethical boards at each study site. Informed consent was obtained from all participants. Sampling weights were generated to adjust for non-response and the population distribution reported by the United Nations Statistical Division.

Data were publicly available for 69 countries. Of these, ten countries were excluded due to a lack of sampling information. Furthermore, ten high-income countries were excluded in order to focus on LMICs. Moreover, Turkey was deleted due to lack of data on PE. Thus, the final sample consisted of 48 LMICs (*n* = 242 952) according to the World Bank classification at the time of the survey (2003). The data were nationally representative for all countries with the exception of China, Comoros, the Republic of Congo, Ivory Coast, India and Russia. The included countries and their sample sizes are provided in eTable 1 in the Supplementary material.

### Subjective cognitive complaints

SCC were assessed with two questions: (a) Overall in the last 30 days, how much difficulty did you have with concentrating or remembering things?; and (b) In the last 30 days, how much difficulty did you have in learning a new task (e.g. learning how to get to a new place, learning a new game, learning a new recipe, etc.)? (Ghose and Abdoul Razak, 2017). Each item was scored on a five-point scale: none (code  =  1), mild (code  =  2), moderate (code  =  3), severe (code  =  4) and extreme/cannot do (code  =  5). Since these answer options were an ordered categorical scale, as in previous WHS studies, we conducted factor analysis with polychoric correlations to incorporate the covariance structure of the answers provided for individual questions measuring a similar construct (Moussavi *et al*., 2007; Nuevo *et al*., 2013; Stubbs *et al*., 2016; Koyanagi *et al*., 2017). The principal component method was used for factor extraction, while factor scores were obtained using the regression scoring method. These factor scores were later converted to scores ranging from 0 to 10 to create a SCC scale with higher values representing more severe cognitive complaints. In order to assess whether there are any differences in the association between the two different types of SCC and PE, we also dichotomised these variables as severe/extreme (codes 4 and 5) or else.

### Psychotic experiences

Participants were asked questions on positive psychotic symptoms (delusional mood, delusions of reference and persecution, delusions of control, hallucinations) which came from the WHO Composite International Diagnostic Interview (CIDI) 3.0 (Kessler and Ustun, 2004) (specific questions can be found in eTable 2). This psychosis module has been reported to be highly consistent with clinician ratings (Cooper *et al*., 1998). Individuals who endorsed at least one of the four psychotic symptoms were considered to have PE.

### Influential factors

We assessed the influence of current smoking, heavy drinking, chronic physical conditions, sleep problems, depression, anxiety, perceived stress and antipsychotic use in the association between SCC and PE for their previously reported association between both cognitive impairment and PE (Tschanz *et al*., 2013; Begum *et al*., 2014; Spira *et al*., 2014; Koyanagi and Stickley, 2015; DeVylder *et al*., 2016; Lara *et al*., 2016; Lara *et al*., 2017; Ballesteros *et al*., 2018; Scott *et al*., 2018). Heavy episodic drinking was defined as having consumed ⩾4 (female) or ⩾5 (male) standard alcoholic beverages on ⩾2 days in the past 7 days (Vancampfort *et al*., 2017). Chronic physical conditions referred to having at least one of arthritis, angina, asthma, diabetes, visual impairment or hearing problems (details on these variables are provided in eTable 3). Sleep problems were operationalised as severe/extreme problems with sleeping, such as falling asleep, waking up frequently during the night or waking up too early in the morning in the past 30 days (Koyanagi and Stickley, 2015). Depression referred to having had a lifetime diagnosis of depression or having had past 12 months depression assessed by questions from the World Mental Health Survey version of the CIDI (Kessler and Ustun, 2004). Anxiety was defined as severe/extreme problems of worry or anxiety in the past 30 days (Koyanagi and Stickley, 2015). Past 30-day perceived stress was assessed with two questions from the Perceived Stress Scale (Cohen *et al*., 1983). The scale in our study ranged from 2 to 10 with higher scores representing higher levels of stress (DeVylder *et al*., 2016). Antipsychotic use was confirmed by the interviewer who checked the drugs used by the participant in the past 2 weeks.

### Control variables

Control variables included those on sociodemographic characteristics (age, sex, wealth, years of education received). Country-wise wealth quintiles were created using principal component analysis based on 15–20 assets depending on the country. We did not assess the influence of these factors in the PE–SCC relationship as these sociodemographic characteristics are often considered to be non-modifiable.

### Statistical analysis

Statistical analyses were performed with Stata 14.1 (Stata Corp LP, College station, Texas, USA). The difference in sample characteristics was tested by *χ*^2^ test and Student's *t*-tests for categorical and continuous variables, respectively. Multivariable logistic regression analysis was conducted to assess the association between SCC (exposure) and PE (outcome). Two models were constructed: model 1 – adjusted for sociodemographics (age, sex, wealth, education and country); and model 2 – adjusted for factors in model 1 and smoking, heavy drinking, chronic physical condition, sleep problems, depression, anxiety, perceived stress and antipsychotic use. In order to assess whether the association between SCC and PE is different by age groups and sex, we conducted interaction analysis by including the product term [age group (18–44, 45–64, ⩾65 years) × SCC] or (sex × SCC) in the fully adjusted model using the overall sample. The results showed that there was no significant interaction by sex but that age is a significant effect modifier. Thus, we also conducted analyses stratifying by age groups. We also repeated this analysis by using the two different types of SCC as the exposure variable.

Next, to gain an understanding on the extent to which various factors may explain the relation between SCC and PE, we conducted mediation analysis using the overall sample. Specifically, we focused on smoking, heavy drinking, chronic physical conditions, sleep problems, depression, anxiety, perceived stress and antipsychotic use. We used the *khb* (Karlson Holm Breen) command in Stata (Breen *et al*., 2013) for this purpose. This method can be applied in logistic regression models and decomposes the total effect of a variable into direct and indirect effects (i.e. the mediational effect). Using this method, the percentage of the main association explained by the mediator can also be calculated (mediated percentage). Each potential influential factor was included in the model individually apart from an analysis where all mental health factors were included simultaneously in the model. The mediation analyses were adjusted for age, sex, education, wealth and country. Finally, in order to assess whether the association between SCC and PE is consistent across countries, country-wise analyses adjusting for age, sex, education and wealth were also conducted.

As in previous WHS studies, adjustment for country was done using fixed-effects models by including dummy variables for each country (Koyanagi and Stickley, 2015; DeVylder *et al*., 2016). All variables were included in the regression analysis as categorical variables with the exception of age, education, perceived stress and the SCC scale (continuous variables). All analyses excluded individuals with a self-reported lifetime diagnosis of psychosis (*n*  =  2424) as PE by definition do not include conditions that reach the clinical threshold for a diagnosis. Some countries were not included in the analyses including perceived stress (Brazil, Hungary, Zimbabwe) and anxiety (Morocco) as data were not collected in these countries. Taylor linearisation methods were used in all analyses to account for the sample weighting and complex study design. Results from the logistic regression analyses are presented as odds ratios (ORs) with 95% confidence intervals (CIs). The level of statistical significance was set at *p* < 0.05 (two-sided).

## Results

**Fig. 1.**
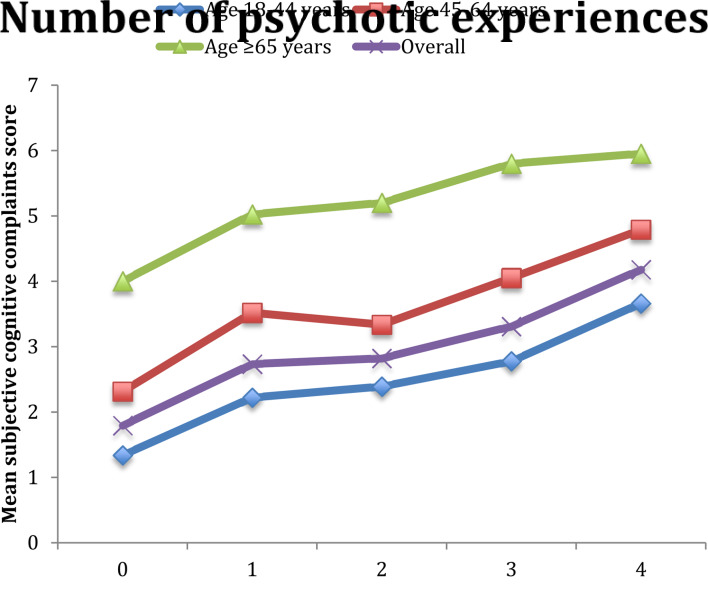
Mean subjective cognitive complaints score by number of different types of psychotic experiences. The variable on subjective cognitive complaints was a scale ranging from 0 to 10 with higher scores representing greater severity of subjective cognitive complaints. The four types of psychotic experiences assessed were: delusional mood, delusions of reference and persecution, and delusions of control, hallucinations

**Fig. 2.**
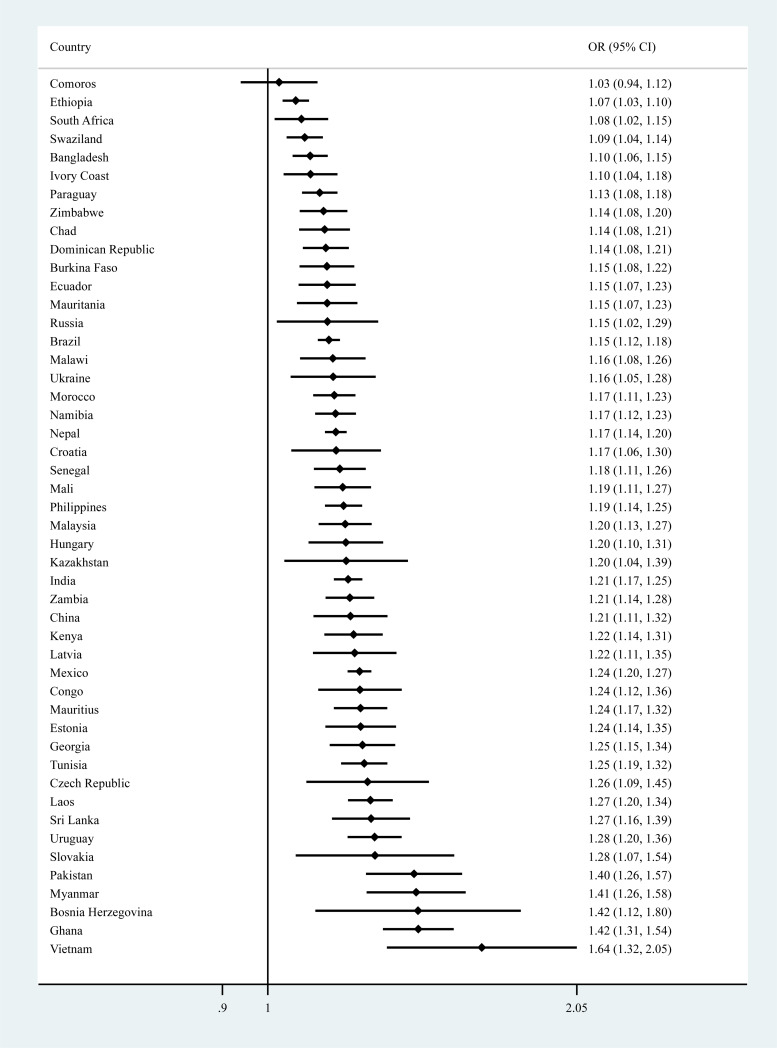
County-wise association between subjective cognitive complaints and psychotic experiences (outcome) estimated by multivariable logistic regression. OR, odds ratio; CI, confidence interval. Models are adjusted for age, sex, wealth and education. The variable on subjective cognitive complaints was a scale ranging from 0 to 10 with higher scores representing greater severity of subjective cognitive complaints.

The final sample consisted of 224 842 people without a psychotic disorder. The overall prevalence of PE was 13.8%. The sample characteristics are provided in Table 1. The mean (SD) age was 38.3 (16.0) and 49.3% were males. There was a near-linear association between increasing numbers of different types of PE and mean SCC scores for the overall sample and all age groups (Fig. 1). In the overall model adjusted for sociodemographics, a one-unit increase in the SCC scale ranging from 0 to 10 was associated with a 1.17 (95% CI  1.16–1.18) times higher odds for PE (model 1) (Table 2). After further adjustment for other factors, the OR was attenuated to 1.08 (95% CI   1.06–1.10) but remained significant (model 2). Similar associations were observed in age-stratified analyses but the association was strongest in the youngest age group. Interaction analysis showed that compared with those aged 18–44 years, the association was significantly weaker among those aged 45–64 years (interaction term *P*  =  0.003) while the interaction term for age ⩾65 years did not reach significance (interaction term *P*  =  0.080) possibly due to lack of statistical power for the small number of individuals aged ⩾65 years (8.5%). The association between PE and the two different types of SCC, when examined individually rather than as a composite score, were similar (eTable 4). The largest proportion of the association between SCC and PE was explained by perceived stress (17.6%), followed by depression (17.4%), anxiety (16.5%), sleep problems (12.3%) and chronic physical conditions (11.8%) (Table 3). When mental health factors (perceived stress, depression, anxiety, sleep problems) were entered simultaneously into the model, they collectively explained 43.4% of the association (data only shown in text). Greater severity of SCC was significantly associated with PE in 47 of the 48 countries included in the study (Fig. 2).

**Table 1.** Sample characteristic (overall and by presence of psychotic experiences)

			Psychotic experiences		
Characteristic	Category	Overall	No	Yes	*P*-value^a^
Age (years), mean ( s.d.)		38.3 (16.0)	38.3 (16.1)	38.2 (15.0)	0.599
Age (years)	18–44	68.0	68.1	67.9	0.007
	45–64	23.4	23.3	24.5	
	⩾65	8.5	8.7	7.6	
Sex	Female	50.7	50.3	53.4	<0.001
	Male	49.3	49.7	46.6	
Wealth	Poorest	20.0	20.0	21.0	0.010
	Poorer	19.9	19.8	21.3	
	Middle	19.9	19.9	19.9	
	Richer	20.1	20.0	19.6	
	Richest	20.2	20.3	18.2	
Education (years), mean ( s.d.)		6.5 (5.2)	6.6 (5.3)	6.0 (4.7)	<0.001
Smoking	No	73.5	74.2	70.0	
	Yes	26.5	25.8	30.0	
Heavy drinking	No	98.0	98.1	97.0	<0.001
	Yes	2.0	1.9	3.0	
Chronic physical condition	No	71.2	73.0	57.0	<0.001
	Yes	28.8	27.0	43.0	
Sleep problems	No	92.6	94.0	83.0	<0.001
	Yes	7.4	6.0	17.0	
Depression	No	89.2	92.4	69.4	<0.001
	Yes	10.8	7.6	30.6	
Anxiety	No	88.9	91.0	74.9	<0.001
	Yes	11.1	9.0	25.1	
Perceived stress,^b^ mean (s.d.)		4.8 (2.2)	4.7 (2.2)	5.7 (2.2)	<0.001
Antipsychotic use	No	99.97	99.98	99.88	
	Yes	0.03	0.02	0.12	<0.001

Data are % or mean (standard deviation).

a *P*-value was calculated by *χ*^2^ tests and Student's *t*-tests for categorical and continuous variables, respectively.

b The variable on perceived stress was a scale ranging from 2 to 10 with higher scores representing higher levels of perceived stress.

**Table 2.** Association between subjective cognitive complaints and psychotic experiences (outcome) estimated by multivariable logistic regression

		Overall		Age 18–44 years		Age 45–64 years		Age ⩾65 years	
Characteristic	Category	Model 1	Model 2^a^	Model 1	Model 2^a^	Model 1	Model 2^a^	Model 1	Model 2^a^
Subjective cognitive complaints^b^		1.17***	1.08***	1.19***	1.10***	1.15***	1.06***	1.14***	1.07*
		(1.16–1.18)	(1.06–1.10)	(1.17–1.20)	(1.07–1.12)	(1.12–1.17)	(1.03–1.09)	(1.09–1.19)	(1.00–1.14)
Age (years)		0.99***	0.99***	0.99**	0.99***	1.00	1.00	1.00	1.01
		(0.99–0.99)	(0.99–0.99)	(0.99–1.00)	(0.98–0.99)	(0.99–1.01)	(0.98–1.01)	(0.99–1.02)	(0.99–1.02)
Sex	Female *v*. male	0.93*	0.98	0.93	0.97	0.98	1.08	0.79*	0.82
		(0.87–1.00)	(0.90–1.08)	(0.85–1.01)	(0.87–1.09)	(0.87–1.11)	(0.93–1.26)	(0.64–0.98)	(0.63–1.06)
Wealth	Poorest	1.00	1.00	1.00	1.00	1.00	1.00	1.00	1.00
	Poorer	1.04	1.14*	1.05	1.14*	1.04	1.15	1.04	1.15
		(0.96–1.14)	(1.03–1.27)	(0.94–1.17)	(1.00–1.28)	(0.89–1.22)	(0.95–1.39)	(0.81–1.33)	(0.87–1.52)
	Middle	0.98	1.09	0.96	1.03	1.03	1.17	1.09	1.34
		(0.90–1.07)	(0.98–1.20)	(0.86–1.07)	(0.91–1.16)	(0.87–1.21)	(0.95–1.44)	(0.80–1.48)	(0.94–1.92)
	Richer	0.99	1.14*	1.00	1.12	1.01	1.14	0.93	1.37
		(0.88–1.11)	(1.00–1.29)	(0.86–1.15)	(0.95–1.31)	(0.86–1.20)	(0.93–1.40)	(0.69–1.26)	(0.96–1.95)
	Richest	0.92	1.09	0.90	1.03	0.98	1.28	0.92	1.11
		(0.81–1.03)	(0.96–1.25)	(0.78–1.03)	(0.88–1.21)	(0.79–1.23)	(1.00–1.64)	(0.67–1.26)	(0.77–1.60)
Education (years)		1.01	1.02***	1.01	1.03***	1.00	1.01	1.00	1.03
		(1.00–1.01)	(1.01–1.03)	(1.00–1.02)	(1.01–1.04)	(0.98–1.01)	(0.99–1.03)	(0.97–1.04)	(0.99–1.07)
Smoking	Yes *v*. no		1.15**		1.16**		1.15		1.21
			(1.05–1.26)		(1.04–1.29)		(0.98–1.35)		(0.94–1.57)
Heavy drinking	Yes *v*. no		1.20		1.16		1.15		1.71
			(0.98–1.48)		(0.91–1.50)		(0.78–1.68)		(0.89–3.28)
Chronic physical condition	Yes *v*. no		1.56***		1.67***		1.39***		1.18
			(1.43–1.69)		(1.50–1.85)		(1.19–1.62)		(0.91–1.54)
Sleep problems	Yes *v*. no		1.37***		1.31**		1.38*		1.73**
			(1.18–1.58)		(1.10–1.56)		(1.06–1.80)		(1.22–2.45)
Depression	Yes *v*. no		2.68***		2.78***		2.58***		2.67***
			(2.33–3.08)		(2.34–3.29)		(2.09–3.19)		(2.04–3.50)
Anxiety	Yes *v*. no		1.66***		1.79***		1.58***		1.34*
			(1.46–1.89)		(1.53–2.11)		(1.26–1.99)		(1.02–1.76)
Perceived stress^c^			1.14***		1.15***		1.14***		1.07*
			(1.12–1.16)		(1.12–1.18)		(1.10–1.19)		(1.01–1.13)
Antipsychotic use	Yes *v*. no		1.90		1.04		1.14		5.64
			(0.57–6.37)		(0.28–3.84)		(0.11–12.06)		(0.66–48.11)

Data are odds ratio (95% confidence interval).

Models are adjusted for all variables in the respective column and country.

a Morocco, Brazil, Hungary and Zimbabwe are not included due to lack of data on perceived stress or anxiety.

b The variable on subjective cognitive complaints was a scale ranging from 0 to 10 with higher scores representing greater severity of subjective cognitive complaints.

c The variable on perceived stress was a scale ranging from 2 to 10 with higher scores representing higher levels of perceived stress.

*p < 0.05, **p < 0.01, ***p < 0.001.

**Table 3.** Mediating effects of potentially influential factors in the association between subjective cognitive complaints and psychotic experiences

Mediator	Effect	OR (95%CI)	*P*-value	% Mediated^a^
Smoking	Total	1.17 (1.16–1.18)	<0.001	NA
	Direct	1.17 (1.16–1.18)	<0.001	
	Indirect	1.00 (1.00–1.00)	0.171	
Heavy drinking	Total	1.17 (1.16–1.18)	<0.001	NA
	Direct	1.17 (1.16–1.18)	<0.001	
	Indirect	1.00 (1.00–1.00)	0.272	
Chronic physical condition	Total	1.17 (1.16–1.19)	<0.001	11.8
	Direct	1.15 (1.14–1.17)	<0.001	
	Indirect	1.02 (1.02–1.02)	<0.001	
Sleep problems	Total	1.17 (1.15–1.18)	<0.001	12.3
	Direct	1.14 (1.13–1.16)	<0.001	
	Indirect	1.02 (1.02–1.02)	<0.001	
Depression	Total	1.17 (1.15–1.18)	<0.001	17.4
	Direct	1.14 (1.12–1.15)	<0.001	
	Indirect	1.03 (1.02–1.03)	<0.001	
Anxiety^b^	Total	1.16 (1.15–1.18)	<0.001	16.5
	Direct	1.13 (1.12–1.15)	<0.001	
	Indirect	1.03 (1.02–1.03)	<0.001	
Perceived stress^c^	Total	1.17 (1.16–1.19)	<0.001	17.6
	Direct	1.14 (1.13–1.16)	<0.001	
	Indirect	1.03 (1.02–1.03)	<0.001	
Antipsychotic use	Total	1.17 (1.16–1.18)	<0.001	NA
	Direct	1.17 (1.16–1.18)	<0.001	
	Indirect	1.00 (1.00–1.00)	0.298	

OR, odds ratio; CI, confidence interval.

Models are adjusted for age, sex, education, wealth and country.

The variable on subjective cognitive complaints was a scale ranging from 0 to 10 with higher scores representing greater severity of subjective cognitive complaints.

a % Mediated was only calculated in the presence of a significant indirect effect.

b Morocco was not included due to lack of data.

c Brazil, Hungary and Zimbabwe were not included due to lack of data.

## Discussion

In our study, we found that greater impairments in subjective cognitive ability were associated with higher odds for PE in adults across the life span and that this association was particularly pronounced in the youngest age group. Perceived stress, depression, anxiety, sleep problems and chronic physical conditions explained 11.8–17.6% of the SCC–PE association (with mental health factors collectively explaining 43.4%) but this association remained significant even after full adjustment. SCC were significantly associated with higher odds for PE in 47 of the 48 countries studied. To the best of our knowledge, our study is the first that specifically focuses on SCC and PE. SCC can identify cognitive changes that affect individuals on a day-to-day basis in contrast to objective cognitive measures, which often do not reflect tasks required for everyday functioning. Importantly, subjective cognitive impairments can be very easily screened in clinical practice by asking patients to self-report recent cognitive difficulties. This stands in contrast to objective neuropsychological measures of cognition, which may be difficult to implement broadly across practice settings (particularly those in which resources or time with patients is limited). Furthermore, subjective cognitive-perceptive basic symptoms have been shown to have high predictive power in identifying subjects at high risk for developing psychosis (Schultze-Lutter *et al*., 2012).

Regarding the age-related trends in the association between cognitive function and PE, this has only been assessed in one study (Mollon *et al*., 2016). This UK study found that medium-to-large impairments in neuropsychological functioning (i.e. IQ, verbal knowledge, working memory, memory) was only observed in the older age group (i.e. ⩾50 years). In contrast, our study found that SCC were more strongly associated with PE in the younger age group. The difference in results may be related to different measures of cognitive function used or with different settings. However, this may also be related with different causes for SCC in the younger and older age groups. For example, one study showed that stress and multi-tasking were frequently reported as the causes of memory complaints among middle-aged adults, while age/ageing was the common cause in older adults (Vestergren and Nilsson, 2011). Thus, it is also possible that SCC are more indicative of mental problems which are not associated with age-related changes in cognition in the younger age group. Our finding that SCC was most strongly associated with PE among young adults who are at highest risk for psychosis supports the potential value of SCC as an indicator for psychosis risk.

The fact that mental health problems explained the largest proportion (collectively 43.4%) of the SCC–PE association may not be surprising given that about two-thirds of PE occur in conjunction with a diagnosable non-psychotic mental disorder, with PE often being a marker of severe affective disorders (Wigman *et al*., 2012; van Os, 2014). Common mental disorders such as depression may also hasten cognitive decline via hypothalamic–pituitary–adrenal (HPA) axis dysregulation and chronic inflammation, which may lead to hippocampal atrophy and frontostriatal abnormalities (Alexopoulos, 2003; Rapp *et al*., 2006; Hermida *et al*., 2012; Diniz *et al*., 2014). In the case of perceived stress, which explained the largest proportion of the association, prolonged elevation of cortisol (HPA axis response to chronic stress) may result in both cognitive decline (Greenberg *et al*., 2014) and PE (Muck-Seler *et al*., 1999). Alternatively, it is also possible that perceived stress due to or complicated by cognitive decline may lead to the emergence of PE (DeVylder *et al*., 2016).

Chronic physical conditions also explained 11.8% of the association. A bidirectional association between chronic physical conditions and PE may exist, although temporally primary PE seem to be more common, and this association may be explained by a complex interplay of factors such as pain, sleep problems and psychological distress (Scott *et al*., 2018). On the other hand, some chronic physical conditions may lead to cognitive impairment via factors such as atherosclerosis, microvascular changes and inflammatory processes (Biessels *et al*., 2006).

The fact that SCC were still significantly associated with PE after adjustment for all potentially influential factors assessed in this study point to the possibility that SCC and PE are linked via other mechanisms. For example, cognitive deficits may lead to PE by directly affecting the way in which events are interpreted (Freeman *et al*., 2011). Furthermore, incipient brain disorders may underlie both cognitive deficits and PE (Stling *et al*., 2004). Finally, genetic factors may also play a role and this is an area for future research (Hatzimanolis *et al*., 2015).

The strengths of the study include the very large sample size and the inclusion of diverse populations across the globe as well as the use of predominantly nationally representative datasets. However, the study results should be interpreted in light of several potential limitations. First, given that all information was based on self-report, there is potential for reporting bias. Second, it is important to note that the aim of the study was to quantify the degree to which potentially influential factors may explain the association between SCC and PE, without differentiating the factors as mediators or confounders. Mediation and confounding are identical statistically and can only be distinguished on conceptual grounds (MacKinnon *et al*., 2000). Third, we lacked data on cannabis use which has been associated with both cognitive impairment and PE (van Gastel *et al*., 2014; Mizrahi *et al*., 2017). However, recent studies have found that PE were associated with cognitive impairment even after adjustment for cannabis use (Mollon *et al*., 2016) and that the link between borderline intellectual functioning and psychosis is not mediated by cannabis use (Hassiotis *et al*., 2017). Fourth, we used two questions to assess SCC. There is no consensus on the measure to capture SCC and the measures used in previous studies range from a single question to a complex assessment involving multiple questions. Thus, the results could have differed if a different measure of SCC was used. Next, previous studies have shown that SCC may have a more psychological component than objective cognitive measures (Homayoun *et al*., 2011), but the association between SCC and PE remained significant even after adjustment for mental health factors such as depression and anxiety in our study. In addition, due to lack of data, we were unable to assess the effect of psychiatric medication such as benzodiazepines or lithium which may be more commonly used by individuals with PE, and may impact on cognition. Finally, the cross-sectional design limits the assessment of temporal association and causality.

In conclusion, our study results showed that SCC were associated with higher odds for PE in adults living in LMICs, and this association was particularly pronounced among younger individuals who are at particularly high risk for psychosis. Future longitudinal studies are warranted to understand the temporal association between SCC and PE, and the utility of SCC in predicting future psychosis risk especially among younger individuals. SCC may serve as a simple and cost-effective screening tool as compared with objective cognitive testing in identifying individuals at high risk for future psychosis onset, pending future research.

## Supplementary material

For supplementary material accompanying this paper visit https://doi.org/10.1017/S2045796018000744. click here to view supplementary material

## Availability of data and materials

The dataset used in this study is publically available to all interested researchers through the following website: http://www.who.int/healthinfo/survey/en/.
