# Supplementary material for: Psychotic experiences and subjective cognitive complaints among 224 842 people in 48 low- and middle-income countries
Source: Epidemiol Psychiatr Sci. 2018 Dec 26;29:e11. doi: 10.1017/S2045796018000744 (PMC8061242; doi:10.1017/S2045796018000744)
Supplement: Supplementary file 1 [file S2045796018000744sup001.docx]

| **eTable 1** Countries included in the analysis and sample size | | | |
| --- | --- | --- | --- |
| Low-income countries | N | Middle-income countries | N |
| Bangladesh | 5,942 | Bosnia Herzegovina | 1,031 |
| Burkina Faso | 4,948 | Brazil | 5,000 |
| Chad | 4,870 | China | 3,994 |
| Comoros | 1,836 | Croatia | 993 |
| Ethiopia | 5,089 | Czech Republic | 949 |
| Ghana | 4,165 | Dominican Republic | 5,027 |
| India | 10,687 | Ecuador | 5,675 |
| Ivory Coast | 3,251 | Estonia | 1,020 |
| Kenya | 4,640 | Georgia | 2,950 |
| Laos | 4,988 | Hungary | 1,419 |
| Malawi | 5,551 | Kazakhstan | 4,499 |
| Mali | 4,886 | Latvia | 929 |
| Mauritania | 3,902 | Malaysia | 6,145 |
| Myanmar | 6,045 | Mauritius | 3,968 |
| Nepal | 8,820 | Mexico | 38,746 |
| Pakistan | 6,501 | Morocco | 5,000 |
| Republic of Congo | 3,075 | Namibia | 4,379 |
| Senegal | 3,461 | Paraguay | 5,288 |
| Vietnam | 4,174 | Philippines | 10,083 |
| Zambia | 4,165 | Russia | 4,427 |
| Zimbabwe | 4,290 | Slovakia | 2,535 |
|  |  | South Africa | 2,629 |
|  |  | Sri Lanka | 6,805 |
|  |  | Swaziland | 3,117 |
|  |  | Tunisia | 5,202 |
|  |  | Ukraine | 2,860 |
|  |  | Uruguay | 2,996 |

| **eTable 2** Questions used to assess psychotic experiences |
| --- |
| Respondents were asked the following questions with answer options ‘yes’ or ‘no’.  During the last 12 months, have you experienced: |
| (i) ‘A feeling something strange and unexplainable was going on that other people would find hard to believe?’ (delusional mood) |
| (ii) ‘A feeling that people were too interested in you or there was a plot to harm you?’ (delusions of reference and persecution) |
| (iii) ‘A feeling that your thoughts were being directly interfered [with] or controlled by another person, or your mind was being taken over by strange forces?’ (delusions of control) |
| (iv) ‘An experience of seeing visions or hearing voices that others could not see or hear when you were not half asleep, dreaming or under the influence of alcohol or drugs?’ (hallucinations) |

| **eTable 3** Assessment of chronic physical conditions | |
| --- | --- |
| Chronic physical condition | Assessment |
| Arthritis | Those who answered ‘yes’ to the question ‘Have you ever been diagnosed with arthritis (a disease of the joints)?’ were considered to have arthritis. |
| Asthma | Those who answered ‘yes’ to the question ‘Have you ever been diagnosed with asthma (an allergic respiratory disease)?’ were considered to have asthma. |
| Diabetes | Those who answered ‘yes’ to the question ‘Have you been diagnosed with diabetes (high blood sugar)?’ were considered to have diabetes. |
| Angina | Those who answered ‘yes’ to the question ‘Have you been ever diagnosed with angina or angina pectoris (a heart disease)?’ and/or those who screened positive on the WHO Rose questionnaire^1^ were considered to have angina. |
| Visual impairment | Visual impairment was defined as having extreme difficulty in seeing and recognizing a person that the participant knows across the road (i.e., from a distance about 20 meters). A validity study showed that this response likely corresponds to World Health Organization definitions of visual impairment.^2^ |
| Hearing problems | The participant was considered to have hearing problems if the interviewer observed this condition by the end of the survey. |

1. Rose GA. The diagnosis of ischaemic heart pain and intermittent claudication in field surveys. Bull World Health Organ. 1962;27:645-658.

2. Freeman EE, Roy-Gagnon MH, Samson E, et al. The global burden of visual difficulty in low, middle, and high income countries. PLoS One. 2013;8(5):e63315.

| **eTable 4** Association between different types of subjective cognitive complaints and psychotic experiences (outcome) estimated by multivariable logistic regression | | | | | | | | |
| --- | --- | --- | --- | --- | --- | --- | --- | --- |
|  | Overall | | Age 18-44 years | | Age 45-64 years | | Age ≥65 years | |
|  | Model 1 | Model 2^a^ | Model 1 | Model 2^a^ | Model 1 | Model 2^a^ | Model 1 | Model 2^a^ |
| Difficulties in |  |  |  |  |  |  |  |  |
| Memory/concentration | 2.63*** | 1.36*** | 2.79*** | 1.47*** | 2.61*** | 1.27 | 2.32*** | 1.51* |
|  | [2.38,2.91] | [1.18,1.56] | [2.42,3.23] | [1.20,1.80] | [2.19,3.10] | [0.99,1.63] | [1.85,2.90] | [1.09,2.11] |
| Learning a new task | 2.19*** | 1.25** | 2.38*** | 1.22 | 2.21*** | 1.32** | 1.95*** | 1.50** |
|  | [1.97,2.43] | [1.09,1.43] | [2.03,2.78] | [0.99,1.50] | [1.88,2.61] | [1.07,1.64] | [1.58,2.41] | [1.12,2.00] |

Data are odds ratio [95% confidence interval].

Variables on difficulties in memory/concentration and learning a new task were dichotomous variables and were included individually in the models.

Model 1 - adjusted for sociodemographics (age, sex, wealth, education, and country)

Model 2 - adjusted for factors in Model 1 and smoking, heavy drinking, chronic physical condition, sleep problems, depression, anxiety, perceived stress, and antipsychotic use.

^a^ Morocco, Brazil, Hungary, and Zimbabwe are not included due to lack of data on perceived stress or anxiety.

* p<0.05, ** p<0.01, *** p<0.001
